# 

**Published:** 1970-07

**Authors:** 


The
Bristol Medico-Chirurgical Journal
(Incorporating the Medical Journal of the South-West)
Established 1883

Vol. 85 (iii) JULY 1970 No. 315

### BRISTOL MEDICO-CHIRURGICAL JOURNAL

Published Quarterly Annual Subscription 16/- Post Free

### BRISTOL MEDICO-CHIRURGICAL SOCIETY

**President 1969-70 : Dr. F. J. W. Lewis**
**President-elect : Dr. Beryl Corner.**
**Honorary Secretary : Dr. I. S. Bailey, 7 Percival Road, Bristol 8.**
**Honorary Treasurer : Dr. Frank Ross, Bristol Royal Infirmary, Bristol 2.**

The Society was founded in 1874. In 1883 publication of the Journal began and has continued quarterly since then. During the years 1949 to 1962 it appeared under the title of " The Medical Journal of the South West ".

### THE BRISTOL MEDICO-CHIRURGICAL JOURNAL

### Editorial Committee

Editor Dr. N. J. Brown, Southmead Hospital, Bristol.

Assistant Editor Mr. J. B. M. Roberts.

Members Dr. M. B. Lennard
Professor R. C. Wofinden
Dr. D. W. Wright
The Hon. Treasurer and Hon. Secretary of the Society

Business Manager Dr. P. J. F. Baskett, Frenchay Hospital, Bristol

Secretary Mr. A. E. S. Roberts, The Library, Medical School, University Walk, Bristol 8

### NOTICE TO CONTRIBUTORS

The Journal is especially concerned to provide a place for the recording of medical thought, interests, and practice in and around Bristol. It therefore welcomes articles that, as well as being of immediate interest to members, will document the local and contemporary medical scene for our successors.

Original articles are invited provided that they have not been published elsewhere. References in the text should be by author's name, with year of publication in parenthesis; they should be listed at the end in alphabetical order, the name of each journal or book being given in full — not abbreviated — and the title of the article added. Illustrations should be supplied ready for reproduction. Line drawings should be in Indian ink on firm white paper or board. The author will be informed if it is found necessary to ask him to pay for the making of blocks.

The following contributions will be especially welcome : notes of forthcoming events, meetings held, degrees conferred, staff appointments, honours and public appointments and other local news.

Twenty-five reprints of his article will be supplied free to each contributor. Quotations for larger quantities will be sent out with proofs and must be ordered when the corrected proofs are returned.

### The Medical Library

In 1890 the Bristol Medico-Chirurgical Society founded a medical library which in 1892 was combined with that of the Bristol Medical School. This became the University of Bristol Medical Library in 1925 and an agreement in that year confirmed the privilege of Members of the Society to use the University Medical Library.

### A Selection of Recent Additions to the Medical Library

ALLAND, A.: Evolution and human behaviour. (Tavistock).

ALLEN, C.: Textbook of Psychosexual disorders. Ed 2. (O.U.P.)

ANDERSON, J.M.ed.: Biology and surgery of tissue transplantation. (Blackwell)

BAIRD, I. M. & HOWARD, A. H.: Obesity. (Livingstone)

BARTALOS, M.: Genetics in medical practice. (Pitman)

BENNION, F. A. R.: Professional ethics. (Knight)

BRAY, P. F.: Neurology in pediatrics. (Wiley)

B. M. J.: Practical psychiatry.

CHESS, S.: Introduction to child psychiatry. Ed. 2. (Grune)

CIBA Collection of Medical Illustrations. Vol. 5, The Heart. Presented by Ciba.

CLAXTON, E. & McKAY, H. A. C.: Medicine, morals and man. (Blandford Pr.)

COOMBS, R. R. A. et al: Recent trends in experimental and clinical allergy. (Karger)

CRAB, H. S. M.: Emergency dental treatment. (Wright). Presented by the publishers.

CROFTON, J. & DOUGLAS, A.: Respiratory diseases. (Blackwell)

DARLINGTON, C. D.: Evolution of man and society. (Allen & Unwin)

DEWHURST, C. J. & GORDON, R. R.: The intersexual disorders. (Bailliere)

EASTCOTT, H. H. G.: Arterial surgery. (Pitman)

EASTHAM, R. D.: Laboratory guide to clinical diagnosis. Ed. 2. (Wright). Presented by the publishers.

ELIAS, H. & SHERRICK, J. C.: Morphology of the liver. (Academic Pr.)

FISCH, C. & SURAWICZ, B.eds.: Digitalis. (Grune)

FOULDS, L.: Neoplastic development. Vol. 1. (Academic Pr.)

FRANCIS, D. C. M. & DIXON, D. W.: Diets for sick children. (Blackwell)

FREEMAN, L. M. & JOHNSON, P. M.: Clinical scintillation scanning. (Hoeber)

FRY, R. J. M. et al: Normal and malignant cell growth. (Springer)

GIRDWOOD, R. H. & SMITH R. N.: Malabsorption. (Edinburgh U.P.)

GREENE, R.: Myasthenia gravis. (Heinemann)

HALL, R. et al: Fundamentals of clinical endocrinology. (Pitman)

HAMBURGER, J. I.: Diagnosis and management of common thyroid problems. (Thomas)

HEALD, F. R.: Adolescent nutrition and growth. (Butterworths.)

HEDINGER, C. E. ed.: Thyroid cancer. (Heinemann)

HILL, Sir D.: Psychiatry in medicine. (Nuffield Provincial Hospital Trust)

HOENIG, J. & HAMILTON, M. W.: The desegregation of the mentally ill. (Routledge)

JACO, E. G.: Patients, physicians and illness. (Free Press)

JANZEN, R.: Pain analysis. (Wright). Presented by the publishers.

KLOPPER, A. & DICZFALUSY, E.: Foetus and placenta. (Blackwell)

KREYBERG, L.: Aetiology of lung cancer. (Universitetsforlaget Oslo)

LILIENFELD, A. M.: Epidemiology of mongolism. (Johns Hopkins Pr.)

MACALPINE, I. & HUNTER, R.: George III and the mad-business. (Allen Lane)

MATHE, G.: Scientific basis of cancer chemotherapy. (Springer)

METTLER, L. E. & GREGG, T. G.: Population, genetics and evolution. (Prentice Hall)

MITCHISON, N. A. et al: Organ transplantation to-day. (Excerpta Medica)

PAULSON, M.: Gastroenterologic medicine. (Lea & Febiger)

Recent advances in forensic pathology. (Churchill)
Recent advantages in orthopaedics. (Churchill)
ROBINSON, R. J.: Brain and early behaviour. (Academic Pr.)
RYLE, A.: Student casualties. (Penguin Pr.)
SINCLAIR, D.: Human growth after birth. (O.U.P.)
SKIPPER, J. K. & LEONARD, R. C.: Social interaction and patient care. (Lippincott)
SMITH, G. H.: Drug dependence. (Churchill)
STEVENSON, I.: The psychiatric examination. (Churchill)
STOLL, B. A.: Hormonal management of breast cancer. (Pitman)
WALTER, W. Grey: Observations on man, his frame, his duty and his expectations. (C.U.P.)
WELLS, P. N. T.: Physical principles of ultrasonic diagnosis. (Academic Pr.)
WILSON, N. L.: Obestity. (Blackwell)

### Bristol Medico-Chirurgical Society

The fifth meeting of the session was held on 11th February, 1970, when Prof. W. J. H. Butterfield, Professor of Medicine at Guy's Hospital Medical School addressed the Society on "Priorities in Medicine".

The sixth meeting, on 11th March, 1970, took the form of a Symposium on Postgraduate Medical Education. Under the chairmanship of Dr. J. E. Cates the speakers were Mr. Huw Griffith, Dr. D. S. Ractliffe and Prof. A. E. Read. A lively discussion ensued.

The subject at the seventh meeting on 8th April, 1970, was "Auto-Immune Disease: Fact or Fiction", the speakers being Dr. John Verrier Jones and Dr. Martin Hartog.

### South-West Orthopaedic Club

The 21st Anniversary Meeting of the Club was held on Saturday, 15th November 1969, in the Medical School of University of Bristol, at which the Third Kenneth Pridie Memorial Lecture was given by Professor R. Merle d'Aubigne of Paris.

### THE PRIDIE MEMORIAL LECTURE

**Joint Reconstruction or Replacement in the Inferior Extremity**

Professor d'Aubigne began by pointing out that joint reconstruction by bone graft or replacement by inert materials were indicated for loss of function and loss of bone substance.

Hip joint problems could always be managed by arthrodesis or generous excision, but it was far better to attempt reconstruction whenever possible. Trochanteric osteotomy could be expected to succeed only when a good joint space with congruous joint surfaces was still present; in more advanced disease the failure rate rose above fifty per cent. One particular indication for trochanteric osteotomy he considered was in avascular necrosis in the young when an amazing process of reconstruction could be expected to follow re-alignment of the necrotic area away from the weight-bearing part of the joint. When the severity of the joint damage excluded osteotomy, replacement of joint surfaces should be considered. In his experience of the use of the Smith-Petersen cup in over one thousand cases, occasionally this method would give unpredictably poor results. It is, however, of particular value in the old acetabular fracture. Replacement of the femoral head by prosthesis could only be expected to succeed if the acetabulum were sound.

In the most severe cases where both joint surfaces are seriously damaged, the Professor considered that total hip replacement arthroplasty was a great advantage and that no other operation gave such consistently good results in these severe cases.

Management of the knee joint posed different problems. Here loss of substance would produce loss of mobility and of stability. Reconstruction by bone graft was only possible using homografts in these large weight bearing joints. In recent years cadaver bone transplants, particularly by Russian workers, have been done with some success. The antigenic properties of the homograft can be lessened by preservation of the grafts at low temperatures. Following his personal experience of fifty cases of cadaver bone transplants, Professor d'Aubigne considered the problems of restoring circulation to avascular bone ends dominates the immunological problems. Failure was often due to the mechanical difficulties resulting from collapse of the avascular graft under stress. The replacement of long sections of shaft bone was more successful, provided good internal support of the graft was maintained by medullary nailing.

The Professor concluded by describing an ingenious reconstructive operation that he had devised for use at the knee. This involved the use of the patella as a vascularised transplant for replacing a femoral condyle or tibial plateau. He had had the opportunity to perform this operation on nine occasions, with pleasing results. Two patients had mobile, useful knees after twenty years, and one patient was still ski-ing nine years later.

**Valgus and Varus of the Foot**

Mr. Dillwyn Evans (Cardiff) said it was necessary to distinguish between feet which are normal in structure but which acquire a deformed position because of muscle imbalance and feet which have a skeletal malformation either because of congenital deformity or because adverse factors influence the development of the growing skeleton unfavourably.

There are two major pairs of malformations of the tarsus which have valgus or varus elements. One pair is cavo-varus with its mirror image, plano-valgus, and the other is equino-varus with its mirror image, calcaneo-valgus. These are two different deformities, though the difference between them may appear blurred when the deformities are mild.

Cavo-varus starts at the heel which, for some reason, develops a varus inclination. If the foot were to remain in line with the heel, weight would be borne only on its outer border; the growing foot feels the need to bring the head of the first metatarsal bone to the ground and it does this by pronating itself around an axis which runs along the shaft of the fifth metatarsal bone. This has the effect of raising the medial longitudinal arch which results in cavo-varus deformity. Conversely, the plano-valgus foot starts with excessive valgus of the heel.

Equino-varus, on the other hand, is a varus displacement of the other tarsal bones on the talus. The navicular is displaced medially on the head of the talus, and the cuboid and os calcis are displaced with it. The heel therefore adopts a varus position, the lateral column of the foot grows longer than the medial column and this in turn produces equinous deformity. Evidence was produced to suggest that equinus in these cases may be caused by the tarsal deformity and that there may therefore be two causes for equinus and not, as has been assumed, one. It has been assumed that the deformity always starts in the calf but it may also start in the tarsus—which would explain why, in club foot, permanent correction of equinus can not be obtained unless the columns of the foot are equalised in length. These deformities are reversed in calcaneo-valgus.

The discussion was opened by Mr. F. C. Dwyer (Liverpool) who briefly outlined his reasons for attempting to correct many valgus and varus deformities of the foot by calcaneal osteotomy.

**Walldius Arthroplasty of the Knee**

Mr. G. Blundell Jones (Exeter) gave a review of eighty insertions of the Walldius artificial knee joint. Flexion beyond 60° was retained or obtained in the majority of cases. Although there had been some complications, including sepsis in seven cases, loosening of the prosthesis in eleven cases but not affecting function, rupture of the patella tendon in one case, and three cases of transient lateral popliteal nerve palsy, the prosthesis had been found to give very satisfactory results in severely disabled patients who were becoming unable to walk. It was not recommended for use where more vigorous activity was required.

The Meeting ended with a panel discussion of three patients with hip joint problems.

A Clinical Meeting was held at the Princess Elizabeth Orthopaedic Hospital, Exeter, on the morning of the 17th November, when with Professor R. Merle d'Aubigne present, a large number of mainly difficult hip problems were presented for discussion.

### News and Notes

### APPOINTMENTS

R. M. Barnes, M.B., Ch.B., Consultant and Deputy-Director, Regional Blood-Transfusion Centre, Wessex R.H.B.

B. R. Silk, M.B., M.R.C.P., Consultant Paediatrician, Kettering General Hospital.

A. M. Wilson, M.B., Ch.B., Consultant Anaesthetist, United Sheffield Hospitals.

### NEWLY APPOINTED CONSULTANT

**Dr. Russell Rees,** M.D., M.R.C.P., has been appointed as Consultant Physician with interest in Cardiology to the United Bristol Hospitals. He qualified at Guy's Hospital in 1951 and held house appointments there and at Brompton Hospital.

After holding various registrar and research posts at Brook General Hospital, Westminster Hospital and Guy's and visiting the U.S.A. to pursue research at Johns Hopkins Hospitals, Baltimore, he became Senior Registrar in Medicine at the Westminster Hospital. His main interest is in cardiology, particularly ischaemic heart disease. He will be living at Winterbourne.

